# Machine Learning Applications for Mass Spectrometry-Based Metabolomics

**DOI:** 10.3390/metabo10060243

**Published:** 2020-06-13

**Authors:** Ulf W. Liebal, An N. T. Phan, Malvika Sudhakar, Karthik Raman, Lars M. Blank

**Affiliations:** 1 Institute of Applied Microbiology, Aachen Biology and Biotechnology, RWTH Aachen University, Worringer Weg 1, 52074 Aachen, Germany; an.phan1@rwth-aachen.de; 2 Department of Biotechnology, Bhupat and Juoti Mehta School of Biosciences, Indian Institute of Technology (IIT) Madras, Chennai 600 036, India; bt15d306@smail.iitm.ac.in (M.S.); kraman@iitm.ac.in (K.R.); 3 Initiative for Biological Systems Engineering, IIT Madras, Chennai 600 036, India; 4 Robert Bosch Centre for Data Science and Artificial Intelligence (RBCDSAI), IIT Madras, Chennai 600 036, India

**Keywords:** machine learning, MS-based metabolomics, metabolic engineering, synthetic biology, metabolic flux analysis, multi-omics

## Abstract

The metabolome of an organism depends on environmental factors and intracellular regulation and provides information about the physiological conditions. Metabolomics helps to understand disease progression in clinical settings or estimate metabolite overproduction for metabolic engineering. The most popular analytical metabolomics platform is mass spectrometry (MS). However, MS metabolome data analysis is complicated, since metabolites interact nonlinearly, and the data structures themselves are complex. Machine learning methods have become immensely popular for statistical analysis due to the inherent nonlinear data representation and the ability to process large and heterogeneous data rapidly. In this review, we address recent developments in using machine learning for processing MS spectra and show how machine learning generates new biological insights. In particular, supervised machine learning has great potential in metabolomics research because of the ability to supply quantitative predictions. We review here commonly used tools, such as random forest, support vector machines, artificial neural networks, and genetic algorithms. During processing steps, the supervised machine learning methods help peak picking, normalization, and missing data imputation. For knowledge-driven analysis, machine learning contributes to biomarker detection, classification and regression, biochemical pathway identification, and carbon flux determination. Of important relevance is the combination of different omics data to identify the contributions of the various regulatory levels. Our overview of the recent publications also highlights that data quality determines analysis quality, but also adds to the challenge of choosing the right model for the data. Machine learning methods applied to MS-based metabolomics ease data analysis and can support clinical decisions, guide metabolic engineering, and stimulate fundamental biological discoveries.

## 1. Introduction

Metabolomics is a rapidly emerging field aiming to identify and quantify cellular metabolites. Together with genomics, transcriptomics and proteomics, metabolomics provides valuable insights into the composition of organisms. Mass spectrometry (MS)-based metabolomics is frequently used because of a wide analyte coverage, high sensitivity, high selectivity and high throughput. Metabolomics raw data are inherently complex, and continuous improvements of analysis pipelines are necessary for optimal information retrieval. The complexity stems from the systemic linear and nonlinear interactions among metabolites and the structure of spectrographic data. The challenges associated with the structure of MS data include features (e.g., peaks) typically outnumbering the samples, high noise levels, batch effects during measurements, and missing values. Hence, the metabolomics community has always been eager to adopt new mathematical and computational tools to improve data analysis. Here, we will focus on the potential of machine learning (ML) to support metabolomics data analysis and show the ability of ML to resolve nonlinear relationships and process large heterogeneous datasets. Moreover, we will focus on supervised ML approaches that provide quantitative predictions and are suitable for hypothesis-driven research [1].

In ML, statistical models are trained on data to make reasonable predictions on unknown data. The ML tools use different algorithms and Table 1 provides a brief overview to commonly used supervised ML models. The ‘overfitting risk’ describes the tendency of a statistical model to fit noise in the training samples, eventually leading to performance losses on the test data. Note, while Table 1 indicates the overfitting tendencies of a ‘typical’ ML tool, each ML tool can be set-up from linear to highly nonlinear. For example, an Artificial Neural Network (ANN) with only linear activation functions is but a linear multivariate analysis, and the Random Forest (RF) will represent complex and possibly noisy relationships when implemented with deep decision structures. The item ‘interpretation’ judges how direct the feature is connected to the target value prediction and thus allows direct biological understanding of the decision. Methods transforming features into latent variables impede the interpretation of individual feature contributions to the prediction. The ‘features/sample’ indicate how robust the ML tools are when there are more features than samples observed, as is common in MS spectrometry. Finally, the ‘implementation’ indicates how easily a running pipeline can be generated reflecting factors such as data processing, and the complexity of hyperparameter choices. The different qualities of the ML tools become particularly exposed when working on diverse data, a topic discussed later.

**Figure 1:**
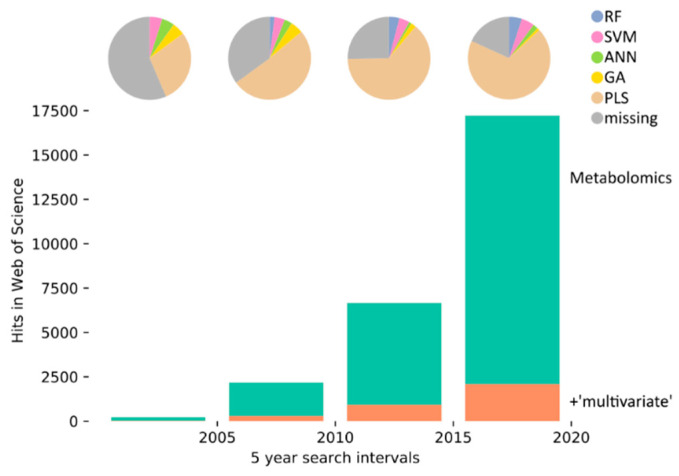
History of machine learning (ML) in metabolomics. The graph shows the frequency of articles mentioning ‘metabolomics’ (green bars) or ‘metabolomics’ and ‘multivariate’ (orange bars) in the Web of Science for five-year intervals from 2000 to 2020. The pie charts represent different statistical analysis approaches, and the absolute number represented by the pie charts is equal to the ‘multivariate’ bar (orange bars). We searched: RF: random forest (blue, ‘random forest’ and ‘decision forest’), SVM: support vector machine (pink, ‘support vector machine’), ANN: artificial neural networks (green, ‘neural network’ and ‘deep learning’), GA: genetic algorithm (yellow, ‘genetic algorithm’ and ‘evolutionary computation’), PLS: partial least squares (brown, ‘partial least squares’ and ‘projection to latent’), and missing (grey). The missing fraction decreases continuously, indicating the adaptation of nomenclature or the conformance of statistical analyses. We searched for ‘multivariate’ to assess the overall number of metabolomics papers with a statistical analysis and obtained similar results for the term ‘statistical’.

Nonlinear data analysis was applied to metabolomics since its origins. Among the first ML methods applied were ANNs: in 1990, Curry and Rumelhart published ‘MSnet’ to distinguish metabolite composition classes [7] and ANNs were continuously applied and improved [8,9,10]. In the late 90s, Genetic Algorithms (GAs) were employed for biomarker identification and to discover interpretable correlations in MS data [6,9]. RF and Support Vector Machines (SVM) entered metabolomics a few years later [11,12,13]. Since then, the frequency of ML-related terms in the metabolomics literature is constant compared to all metabolomics articles (Figure 1). Publications on metabolomics have increased explosively since 2000, with currently over 17,000 publications (Figure 1, green bar). While articles with ML tools grew with a similar rate compared to the overall metabolomics articles, the analytical approach of projection to latent structure (aka Partial Linear Regression, PLS) increased even stronger and dominates the metabolomics analysis. Among the ML tools, the popularity of GAs dropped while RFs experienced the largest overall growth, and ANNs grew particularly during the most recent period.

ML has made rapid progress and now supplies a diverse spectrum of algorithms. Most of the ML tools developed have user-friendly interfaces and enable chemometricians to test various ML solutions and improve the applications for spectral analysis. For example, the Scikit-learn environment in Python provides functions for implementation, along with excellent documentation [14]. The Python library TPOT is an ML support system with an automated generation of tree-based predictors [15]. MS data analysis guides and add-ons for tools with a visual interface were published for WEKA [16] and KNIME [17]. As mentioned, ANN-based analysis is rapidly evolving. Many data analysis fields have embraced ANN, fueled by the availability of large datasets, hardware developments and the development of novel algorithms. New methods evolved from ANN, including convolutional neural networks (CNNs) suited for peak characterization and encoder–decoder systems suited for latent variable projections. Several software tools help the implementation of ANN-based data analysis, for example, Keras with TensorFlow implementation, Theano, Caffe, or Pytorch [18]. Additionally, the unabated interest in ANN produces a vast source of educational material and ‘massive open online courses’ (MOOC).

**Figure 2:**
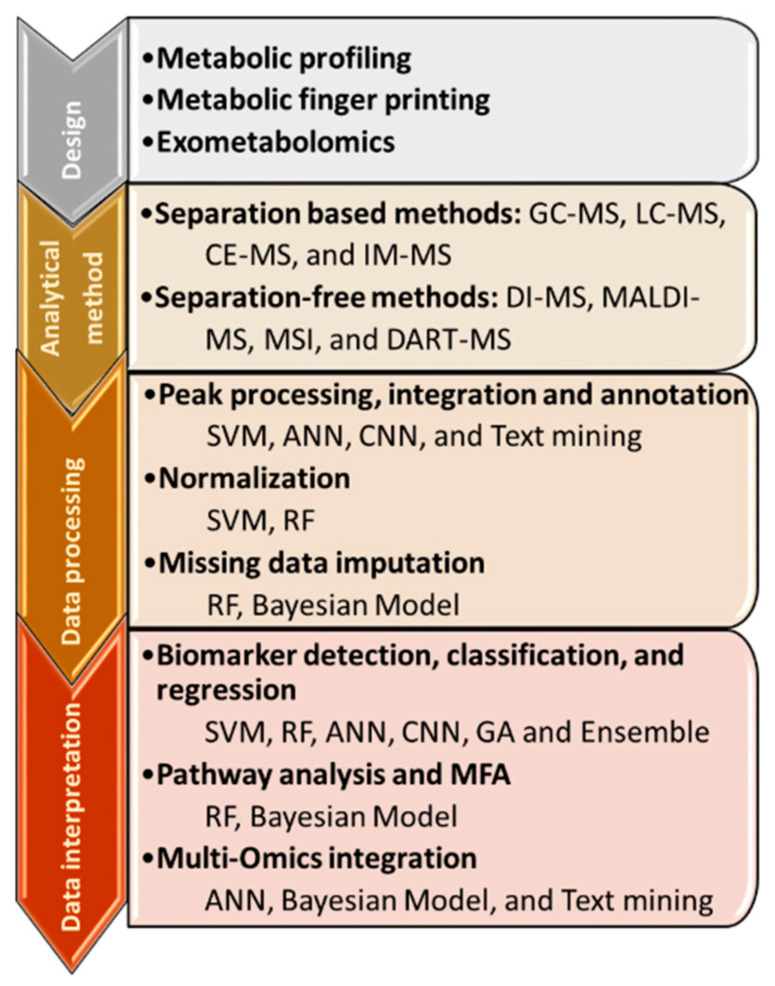
Mass spectrometry workflow with technical and analytical techniques. The MS investigation begins with the definition of the design of the experiment, whether a comprehensive metabolic overview is required, metabolite class identifications are sufficient or targeted metabolites are quantified. The design determines the analytical methods that are distinguished by their metabolite separation. The data processing includes peak processing, normalization and imputation and the contribution of machine learning is discussed in Section 2. The data interpretation is covered in Section 3 and deals with classification and regression, pathway analysis and multi-omics integration. Abbreviations: GC: gas chromatography; LC: liquid chromatography; CE: capillary electrophoresis; IM: ion mobility; DI: direct infusion; MALDI: matrix-assisted laser desorption ionization; MSI: mass spectrometry imaging; DART: direct analysis in real time.

In this review, we give a summary of the recent developments of supervised machine learning for metabolomics analysis. Specifically, we focus on random forest (RF), support vector machine (SVM), artificial neural network (ANN), and genetic algorithm (GA). Figure 2 shows the metabolomics workflow and the steps benefitting from ML. First, we discuss the procedures of data processing that have benefitted from ML, including peak picking and integration, metabolite annotation, data normalization, and imputation. We continue to examine data to knowledge processes, including biomarker detection and classification, metabolomics–ML pathway analysis, interactions with mechanistic models, and multi-omics integration. We conclude by highlighting the need for standardization and benchmarking of ML applications for their efficient dissemination.

## 2. Machine Learning for Data Processing

Data processing, including baseline correction, noise filtering, peak detection, and alignment, is used for precise metabolite annotation and quantification [19]. There are more than 120 million compounds available in the universal compounds databases such as PubChem [20], ChemSpider [21], or the Chemical Abstracts Database with approximately 1–2 million compounds with biological relevance [22]. Peak annotation is among the biggest obstacles in metabolomics because less than 20% of the detected peaks were annotated in non-targeted metabolomics [23]. For the metabolites cataloged in databases (Table 2), annotation is accurate and efficient (reviewed by [24]). Since the mass spectra from structural isomers could be very hard to distinguish, it is crucial to verify the library search results with a reference chromatographic retention and spectrum of the authentic standard [25]. On the other hand, the identification of ‘unknown’ metabolites is challenging due to the lack of commercial standard compounds. Nowadays, many steps of data processing are provided by vendors of analytical equipment. In addition, there are several software tools for manual data processing, as reviewed by O’Shea and Misra (2020) [26].

Prior to statistical analyses, all data should be normalized to exclude sample-to-sample variations, especially when integrating results from different batches or different analytical instruments. In the following, we discuss the different data processing steps, including (i) peak picking and integration, (ii) metabolite annotation, (iii) normalization (incl. scaling), and (iv) missing data imputation. Data scaling or transformation are frequently used to adjust biases among various metabolites and to reduce heteroscedasticity in high-dimensional metabolomics data [27]. Afterwards, multivariate analysis methods are frequently used to get a general view of the dataset and to reveal the relevant metabolites. During model development, overfitting can limit the predictive capacity, and cross-validation is always required [2,28]. After data processing, the final data can be used to get new insights into biological processes.

### 2.1. Peak Picking, Integration and Annotation

Several ML-based approaches were developed to process and select chromatogram peaks (Table 3). Peaks have a strong local correlation, and convolutional neural networks (CNNs) are popular to process, select and integrate peaks [42,43]. Peak picking can be improved if the retention time is known. The ab initio prediction of metabolite retention time is a complex problem because unknown subsets of metabolite atoms are involved. In the first step of retention time prediction, the structural information is encoded in a vector format, e.g., with a quantitative structure–retention relationship (QSRR) [44,45] or molecular fingerprints [46]. Bouwmeester et al. (2019) [47] conducted an illustrative comparison of different ML approaches for LC retention time prediction. The authors extracted 151 features from the SMILES notation to train seven linear and nonlinear models and found best performance for ensemble approaches of combinations of multiple ML tools, while ANN and SVM also performed well alone [47]. Surprisingly, for retention time prediction based on molecular fingerprints, ANNs were only marginal better compared to selecting a retention from the most similar known fingerprint [48]. While data preprocessing increases the information content of the raw data and allows for more complex analysis, methods were developed to bridge from raw spectral data directly to phenotype characterization. Zhang et al. [49] used unprocessed m/z spectra and trained a CNN model called ‘DeepSpectra’ for single-value regression outputs like biomass or protein content from targeted metabolomics of environmental samples.

We give a brief update adding to recent illustrative reviews on ML-assisted metabolite annotation, including fragmentation prediction [23,50]. A prerequisite for molecular structure-based analysis methods is the conversion of the chemical structure into a molecular fingerprint in the form of a standardized vector with binary elements and defined length. The mutual conversion of spectrum and fingerprint started with SVM [51] and eventually developed as the ‘SIRIUS’ annotation tool [52]. We illustrate the benefit of recently published neuron-based ML tools with two examples: (i) the prediction of MS spectrum from fingerprints by ‘NEIMS’ and (ii) the prediction of fingerprints from MS spectrum by ‘DeepEI’. Starting from the fingerprint of the 2017 NIST Mass Spectral Main Library, ‘NEIMS’ predicts the MS spectrum for pure metabolites [53]. The fingerprint vector is non-local: neighboring vector elements code for different chemical properties while distant elements can encode similar properties with respect to MS fragmentation. On non-local feature vectors, ANNs perform well because the hidden nodes connect all vector elements to identify predictive combinations. In contrast, the spectral information is local and CNNs excel in the analysis. Thus, the CNN approach in ‘DeepEI’ tackles the reverse challenge, to predict fingerprints from spectrum, and indeed both strategies can be combined [54]. A new approach used text mining to associate fragmentation groups with metabolite candidates. The underlying assumption is that re-occurring peak patterns represent coherent substructures and can be associated to published metabolite spectra [55,56].

### 2.2. Normalization Procedures

For high-quantity samples, metabolite-specific degradation dynamics and instrument sensitivity declines lead to nonlinear signal variations. Quality control/quality assurance (QC/QA) samples measured throughout the analysis are used to exclude inter- and intra-batch variations while preserving biological information. ML-assisted normalization methods mainly employ SVM- and RF-based regression. Normalization based on SVM has shown mixed performance. SVM outperformed linear and polynomial regression for PCA; however, OPLS-DA showed clear signs of overfitting [68]. The limitation of most existing QC-based normalization methods, including polynomial regression and SVM, is the underlying assumption that the systematic error in each variable is only associated with the batch effect, the injection order, and/or processing sequence. Fiehn and co-workers additionally accounted for error correlations between compounds using the normalization procedure: systematic error removal using random forest (‘SERRF’) [66]. This method assumed that the intensity drift of a metabolite can be summarized and predicted by batch effects, injection orders, and intensity drifts of other compounds. During a comprehensive comparison of normalization methods, ‘SERRF’ outperformed all other existing methods, including SVM and polynomial regression, and significantly reduced the average technical errors to 5% relative standard deviation. Subsequent multivariate analysis, including PCA and PLS-DA, revealed a biological variance of interest without overfitting. Yet, the authors also suggested that ‘SERRF’ performance may vary or not be necessary for small datasets (fewer than 500 samples).

If quality control samples are absent, they can even be simulated from the data. The procedure is performed by ‘pseudoQC’ with the goal to reduce data variation [67]. SVM, RF and linear models were trained on data with low variation but only the nonlinear methods decreased the variation in the test data. A subsequent PCA indicated optimal separation by the RF normalization and was recommended by the authors as the first-choice method for metabolomics data by ‘pseudoQC.’ Together, all reports reached the same agreement that nonlinear regression methods are more appropriate than linear methods for quality-control based normalization to remove batch effects of large-scale metabolomics data. Although SVM and RF have been examined only in a few applications, RF seemed to perform better while dealing with overfitting. Nevertheless, sample size and the number of quality control samples influenced the performance of the normalization process, and further investigations are needed. In summary, for normalization several methods should be tested, while choosing for the best trade-off for local peak properties, like standard deviation, and the performance of subsequent multivariate analyses.

### 2.3. Missing Data Imputation

MS-based analytical methods have a significant advantage in metabolite coverage, but a significant proportion of data are missing values. Notably, LC–MS missing data could be even in the range of 30–50% [71,72]. Different types of missing data are classified. In most cases, data are missing not at random (MNAR) owing to real absence of the compound in the samples or peak detection failure of low-concentration metabolites. There are two other types of missing data, including missing at random (MAR) and missing completely at random (MCAR). While MAR is usually caused by a failure in data preprocessing, such as inaccurate peak detection and deconvolution of co-eluting compounds, MCAR is mainly due to the data acquisition process like incomplete derivatization or ionization [73]. Data imputation is the procedure using the information of existing data to substitute the missing values without changing the whole data structure.

The imputation of missing values is necessary because most statistical data analysis approaches cannot process null information and a reasonable imputation strategy introduces less bias compared to feature removal. Various strategies exist to replace missing values with realistic estimates; however, the optimal strategy depends on the missing value type and also on the subsequent statistical analysis. Thus, the ability to discriminate between the missing value origins is advantageous, although often not known a priori. Shah et al. [70] used a Bayesian model to first discriminate between random and not random missing data, and to sample an imputed distribution using a Markov chain Monte Carlo procedure. Independent of the Bayesian model approach, the best performing procedure was achieved with random forests [69]. Random forest performs best for MAR and MCAR, in combination with subsequent multivariate statistics, like PCA [74]. ANN was inferior to RF, the computation time was inadequately high, and each imputed data needed training of a dedicated ANN estimator with limited data [75]. Imputation is fundamentally a statistical problem; an appropriate sample is taken from the statistical distribution of a metabolite; hence, ML methods are unlikely to replace linear statistical approaches.

## 3. Biological Insights with Metabolomics

In this section, we will discuss various topics for data analysis such as biomarker detection, classification and regression, pathway inference, the combination with mechanistic models, and multi-omics integration. The results will testify to the impressive predictive capacities of ML approaches, but will also reiterate that there is no predefined route to data analysis. Our examples provide broad coverage of the field; for more clinically oriented ML-assisted metabolomics analysis; see the review by Lee and Hu [76]. The ML approaches are statistical methods and thus extract statistical information from the data: their underlying question is: ‘who correlates with whom?’ In the following sections, we will explore the extent to which ML models were used to gain knowledge.

Given the multitude of ML approaches, we are provoked to ask: ‘are there guidelines to select appropriate ML approaches?’ The following sections will reveal the complexity of the question, and it is instructive to clarify the relations among the different ML approaches to judge their performance and requirements. The PLS approach is fundamentally an ANN with one hidden node and linear activation functions [77]. The nonlinear SVM (e.g., with RBF) is similar to an ANN with a single hidden node and nonlinear activation function (e.g., with ReLU). The SVM applies the nonlinearity directly on the variables, whereas the ANN acts on linear variable interactions (the latent variables) [78]. The GA resembles a sparse ANN with more complex and diverse activation functions and the use of evolutionary strategies to improve. By contrast, the ANN uses appointed functions for smooth analytical, gradient-based optimization (backpropagation). The RF is conceptually different and cannot be interpreted in a formulaic way; see Table 1 for a brief description. An overview of articles with ML-assisted metabolomics analysis published since 2019 is given in Table 4. The majority of articles use multiple ML methods for data analysis and usually recommend the optimal algorithm. Overall, however, each ML approach is recommended eventually, even for comparative studies with diverse datasets no definite front-runner can be nominated.

### 3.1. Biomarker Detection, Classification, and Regression

We start by introducing the concepts of this section with an illustrative example of the microbes and metabolites in the digestive system. Two studies on the relation of gut microbes and ambient metabolome reveal how microbes predict metabolite concentrations, and how the latent variables of an ANN provide interpretable biological information. The data sources combined are metabolite feature concentrations and microorganism abundance. Le et al. [92] trained the microbe–metabolite relation into an ANN with an encoder–decoder network. The microbe abundance was used as the input and was mapped to a hidden layer, the latent variables, with a lower number of nodes to represent microbial interactions. The latent variables generated the metabolite levels on the output, and, interestingly, the latent variables contained clinically relevant information to discriminate bowel disease conditions [92]. Morton et al. [93] used a neural network called ‘mmvec’ for analyzing the co-occurrence of microbe–metabolite pairs. The approach can deal with compositional data, i.e., relative concentration level, and data of different magnitudes in general. The method is broadly applicable and was tested over a diverse set of microbiome benchmark datasets including soil biocrust, lung mucus and digestive tract. The importance of the transformation method for scale invariance during preprocessing of microbe–metabolite data was pointed out by Quinn and Erb [96]. The selection between joint and independent probabilities of the bacteria determines the normalization parameter, which biases the performance of the linear estimators in the comparison [97]. The examples testify to the capacity of ML tools to serve biomarker detection, classification, and regression, and furthermore remind us about the complexity of the data for which we need to find suitable preprocessing strategies.

While metabolomics data are dense, the information-rich features are only a small subset of all detected features; moreover, the features frequently outnumber the sample size greatly. With too many features, training of the ML algorithm takes longer, the risk of overfitting increases, and model interpretability is compromised. Feature selection or feature extraction are dimensionality reduction strategies to alleviate the dense data problem (Table 5). Feature selection describes methods that pick features with the highest information and is generally useful for biomarker detection. Feature extraction transforms the features into lower-dimensional latent variables. While retaining most of the information, however, the latent variables generated by feature extraction are difficult to interpret because they have no direct biological counterparts [98]. Feature extraction is useful when the features are not correlated, and each feature is informative. When doing nonlinear data analysis, it is advisable to refrain from overusing linear-based feature selection methods, like regularization, or Linear discriminant analysis (LDA), because they remove the nonlinear features of complex interactions [98]. Particularly useful dimensionality reductions for ML are Recursive Feature Elimination (RFE) for SVM, or encoder–decoder systems for ANN. A related problem to dimensionality reduction is the identification of the most predictive features for classification, ultimately resulting in biomarker detection, a topic excellently reviewed by Xia et al. [99].

The sample size is an important parameter that determines how well statistical interactions can be resolved, and detailed guidelines are available for spectrographic experiments [100]. Typical metabolomics sample sizes are in the range of hundreds, with some below fifty and some over one thousand (Table 4). On the lower limit, one study reported robust binary classification with as little as three samples in each class for linear SVM with untargeted data derived from archaeal cultivation and pig urine after traumatization [101]. ANN performed surprisingly well in a comparative analysis, even with 46 case and 56 control samples in a targeted LC–MS analysis with 42 metabolic features [78]. Similarly, GAs were used for three-class classification with just 20 samples per class and 2700 metabolomics features detected in a high-resolution fingerprint analysis [85]. In this study, the GA approach outperformed RF, probably due to the large potential feature number over the small sample set. The problem of RF to deal with dense data with few informative features was also documented by Mendes et al., 2019 [78], and the data hunger of RF compared to SVM and ANN was previously identified [102]. Note that in Table 4, RF is only competitive in a study with Lasso-regularization of the data, resulting in a rather linear problem with 703 samples [82]. Overall, Table 4 demonstrates the practicality of ML approaches even for small sample sizes. However, not only the sample size is important, but also data quality.

Binary classification problems are often simple enough that conventional statistical approaches outperform machine learning. Mendez et al. (2019) [78] tested eight different linear and ML approaches for their performance in binary classification on ten clinical datasets from targeted metabolomics. Unsurprisingly, the classification results depend more on the data than the applied algorithm. However, crucially, linear classifiers performed similar to SVM or ANN in the majority of datasets. While overall SVM performed best and ANN nearly equally well, RF performed overall poorly—apparently the problem was linearly separable, and only a small fraction of features contained relevant information. Not all binary classifications are linearly separable, as Morais et al. [79] tested on datasets from untargeted infrared spectroscopy with differing covariance using LDA, QDA, and SVM. Only for an evenly distributed variance and correlating covariance was LDA competitive to QDA and SVM.

Each ML tool applies a distinct strategy for statistical analysis and yields best performance when fit to appropriate data structures. These data structures include frequency distributions or data types like canonical or linear data, connected or independent data, which are often not known in advance. Because each dataset is unique, and any data property can affect the performance of the different statistical approaches, it is advisable to test multiple ML tools on the data. Notably, linear multivariate analysis approaches like PLS need to be included as many reports showed their competitiveness. The crucial consequence is that any model is just as good as the data, and careful experimental design remains the strongest indicator for a good model [103].

### 3.2. Metabolomics to Pathways

ML is excellently positioned to analyze metabolomics data and has provided impressive predictive competencies, but the knowledge gain, in general, is limited. The biological and chemical disciplines preferably use mechanistic models to enable the testing of hypotheses and extrapolation to experimentally inaccessible regimes. The most popular mechanistic models for metabolomics data analysis are kinetic models and stoichiometric constraint-based models. The integration of ML with constraint-based models was recently discussed [104,105]. Kinetic models can directly represent metabolite concentration data to predict general properties like metabolic stability, sensitivities as well as dynamic concentration changes. The most considerable disadvantage of kinetic models is the need for substantial knowledge about enzymatic kinetic parameters, restricting their application to small systems, particularly for signaling and regulation [106]. However, ML-based approaches are being developed to alleviate the parametric bottleneck and to support mechanistic model formulation [107,108].

The ability of ML to predict pathway properties based on targeted metabolite information has contributed to improving strains in metabolic engineering. Costello and Martin [109] simulated metabolite dynamics by using metabolite and enzyme concentrations as input to predict the concentration change to the next time-step to identify enzyme contributions to enhance limonene and isopentenol production. They showed that with as few as two strains, the model was capable of extrapolating reasonable dynamics. The procedure is based on the automated ML-pipeline ‘TPOT’ with various data processing steps, linear statistics- and tree-based methods [15]. Other studies use ANN to estimate the effect of gene expression factors when a complete characterization is combinatorically infeasible. For example, finding the optimal ribosome binding site sequence for multiple recombinantly expressed enzymes is experimentally demanding because a large sequence space needs to be tested. However, testing less than a hundred combinations allowed the ANN to derive a sequence that significantly increased production of industrial relevant metabolites [87,110]. An alternative target is promoter activity that was screened for increased productivity [111].

During pathway enrichment, metabolomics data are interpreted in the biological context to identify active pathways. Pathway reconstruction is typically performed with genomic information of cataloged enzyme activities and represents the general metabolic capabilities of an organism. With metabolomics, pathway activities represent conditions after post-translational effects, like enzyme modifications or allosteric regulation, thus providing much more representative information compared to genomics or proteomics approaches. Current statistical approaches include MetScape or Mummichog [112]. A comparison of several tools for metabolite correlation network construction was performed by Jahagirdar et al. [88]. The test data were simulated with a kinetic model of the arachidonic acid degradation pathway and comprised 500 samples with 83 metabolites. The results showed an advantage of RF methods and Bayes models over linear statistical approaches.

Toubiana et al. [89] used an RF to predict active pathways from metabolite correlation networks. The authors associated metabolites and pathways and used the measured metabolite correlations to calculate feature vectors based on metabolites for each pathway using statistical, graph- and correlation network-related metrics. The RF was trained to classify activity from the feature vectors of 169 organism-related active pathways from the MetaCyc databases, 85 non-active pathways, and 85 random metabolite combinations. The approach is limited to the identified metabolites and the predefined pathways for which the training was performed [89]. Hosseini et al. [90] weighted the activity of a pathway by the likelihood that the metabolites are connected to the pathway. The authors constructed a generative model that links pathway activity probabilities to metabolites and eventually to measured spectral masses. Because the tool emphasizes metabolites that are unique for a pathway, the predictions differ from standard enrichment analysis.

Metabolic flux analysis (MFA) based on targeted metabolomics of labeling experiments allows an understanding of metabolic network properties. In MFA, the accumulation of ^13^C isotopically labeled substrates within the metabolites, in combination with cellular physiology, allows for computing intracellular metabolic rates and global flux distributions [113]. Machine learning has so far supported MFA in two directions: (i) an analytic-based surrogate model and (ii) similarity-based flux identification. The analytic-based surrogate model by Kogadeeva and Zamboni [114] is based on flux ratio analysis, and a stoichiometric metabolic model with flux constraints is used to simulate thousands of surrogate labeling distributions. Regression with a random forest procedure associates the surrogate labeling data as input to the associated flux ratios. The approach is context specific to the network used to generate the flux ratios, and the concept can be regarded to accelerate the identification of realistic cellular flux distributions. While the ‘SUMOFLUX’ approach directly supports the flux prediction from label information, the similarity-based flux identification by Wu et al. [115] is an alternative to flux identification with constraint-based linear optimization. ‘mflux’ is an SVM-based regressor and combines one-hundred measured flux distributions of different organisms. A web interface can be used to generate likely central carbon flux distributions based on just ten features like species, reactor type, and nutrient conditions. Metabolic flux analysis requires detailed mechanistic models to understand labeling patterns, and therefore ML approaches with their un-mechanistic functions will instead take a supporting role.

### 3.3. Multi-Omics Integration

Studies are no longer limited to a single omics level with the advent of increasingly faster and cheaper high-throughput technologies. The integration of multiple omics levels will enhance our understanding of the interactions among the different biological layers. The review by Noor et al. [116] gives an overview of the different data-based and knowledge-based methods available for multi-omics integration. In this section, we review the contributions of ML to the integration of multi-omics datasets and the tools available for metabolomics analysis along with the insights obtained. We conducted a general morphological analysis and defined various categories relevant to the research of multi-omics data integration [117]. The categories were used to construct the cross-consistency matrix (CCM) (Table 6), where each cell contains references to studies exploring the categorical research space and blanks reveal potential areas to explore and analyze in the future.

**Figure 3:**
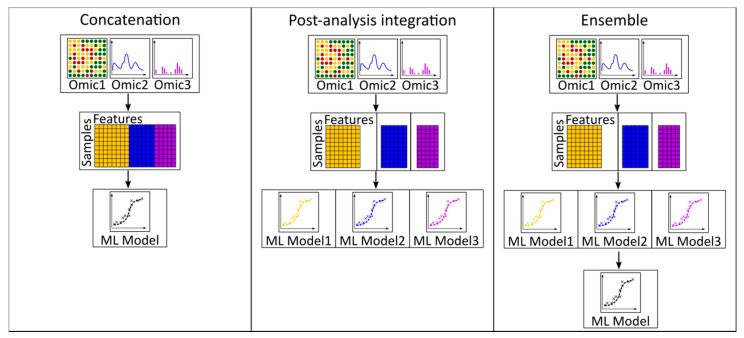
Strategies for multi-omics integration. Omics data can be combined in a single matrix with all omics features, called ‘concatenation,’ or each omics measurement is separately analyzed, called ‘post-analysis integration,’ or the data is concatenated, but instead of a single ML model, many models are trained and their results are combined to calculate the optimal response, called ‘ensemble.’.

We defined five categories, namely, ‘data,’ ‘model,’ ‘integration method,’ ‘dimensionality reduction,’ and ‘model organism.’ The ML approaches used for analysis are listed under the category ‘models’ (Table 6). Since metabolic analysis is mostly constrained to model organisms, this category gives an overview of the published work. The method of integration differed among multi-omics studies and was classified into three subcategories (Figure 3). The most common method for integration is ‘post-analysis,’ in which each omics level was individually analyzed, and the results were only subsequently correlated to understand the mechanism of regulation between each level. An ‘ensemble’ method modeled each omics level separately, and the weighted models are used to make the final predictions. ‘Concatenation’-type integration simply concatenated the different omics features into one feature vector and was analyzed by a single model. Integrating data using concatenation and ensemble methods discovers data correlations across omics layers that are invisible to the post-analysis approach. The post-analysis, however, is relevant for analyzing data from different experiments when homogeneous data across omics sets are not available.

Multi-omics integration increases the number of features with the addition of each omics level, stressing the importance of dimensionality reduction. Cellular features are highly correlated, and models assuming feature independence might perform poorly. Acharjee et al. [118] used RF models to integrate metabolomics and lipidomics to predict clinical phenotypes and drug dosage. They observed prediction improvements after dimensionality reduction on the integrated omics dataset. Similarly, Manor et al. [119] used an RF to predict the plasma level of a disease biomarker with protein, metabolite, and taxonomic features from the gut microbiome. Features ranked by the RF model built only on clinical and microbiome data were compared to highly correlated features. The RF model identified highly correlated features as well other novel features reported in other studiesand including other omics data enhanced biomarker prediction [119]. Moreover, multi-omics integration improved single-omics models for biomarker discovery [120] and disease identification [121].

Multi-omics analysis is more potent if mechanistic knowledge is used to connect the biological layers, a procedure well suited for Bayesian models. The Bayesian model ‘iSchrunk’ samples metabolite concentrations based on kinetic parameters and served to generate surrogate samples for training an RF-like classifier to estimate control coefficients [122,123]. A Bayesian approach with linlog kinetics was used by St John et al. [94] to integrate metabolomics and enzyme concentration levels. The model allowed detailed metabolic characterization, including control coefficients to guide rational strain engineering. A Bayesian-type model was used by Liebermeister [95] to estimate combinations of enzyme kinetic properties, thermodynamics, metabolite and enzyme concentrations, and intracellular fluxes based on linear programming. An approach by Heckmann et al. [107] applied an ensemble of models to elucidate enzyme kinetic parameters. The inputs were enzyme biochemical and structural properties with network-based features to predict the enzyme turnover rates. The rates were used to parameterize a genome-scale model with metabolic and gene expression reactions and resulted in an improved representation of proteome data. The studies show the feasibility of generating large-scale dynamic models with reasonable kinetic parameter estimates.

Many tools integrating multi-omics datasets have been published and implemented in other research areas with the potential to be used with metabolomics data. ‘AutoOmics’ finds ANN for each omics layer and converts the input into the latent variables. The final layers from each omics technology are concatenated and used to train a final ANN model. ‘MixOmics’ is an R package with tools for univariate, multivariate, and multi-omics analysis. Other tools use matrix decomposition [124,125], graph-based methods [126,127,128], or integrate the omics data into genome-scale metabolic models [129,130]. Overall, if enough data is available, ANN and RF methods are well suited to capture nonlinearity and provide interpretability to understand the biological context.

## 4. Conclusions and Outlook

With an unprecedented accumulation of information, the relevance of machine learning intensifies and new algorithms and tools mushroom. According to the No Free Lunch Theorem, no general best-performing optimization algorithm can exist and thus there will always be competing algorithms streamlined to sets of special problems [138]. While no one ML method is better than the other, the model selection and performance depends on data properties and the experiment objective. Thus, standardization and benchmarking are important. The Metabolomics Society proposed the Metabolomics Standards Initiative (MSI) with community-agreed reporting standards, regularly used as a publication requirement in peer-reviewed journals [139]. With an accelerating output of new methods, the development of benchmark datasets becomes urgent. This is challenging because the benchmark sets need to be widely accepted and representative of the data diversity in the field. However, once available, benchmarks form the basis for a comparable documentation of statistical advances and suitable data properties for new methods. These methods, tailored to technological advances boosting data quality and quantity, will contribute to extract the full potential from metabolomics: to guide clinical decisions and deepen our knowledge of metabolism.

## Figures and Tables

**Table 1:** Description of important supervised statistical models.

Supervised ML Model		Advantage	Disadvantage
PLS—Projection to Latent Structure/Partial Linear Regression [2]			
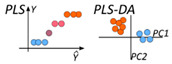	PLS is a supervised method to construct predictive models when the factors are collinear. PLS-DA is an extension of PLS that can maximize the covariance between classes. Orthogonal PLS (OPLS) is an extension to increase latent feature interpretability.	Overfitting risk: Low Interpretation: High Features/sample: High Implementation: Easy	Collinear data
RF—Random Forest [3]			
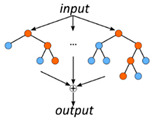	Composed of several decision trees. Each decision tree separates the samples according to the measured feature properties. Different trees use a random subset of samples and features for classification.	Overfitting risk: Medium Interpretation: High Implementation: Easy	Features/sample: Low
SVM—Support Vector Machine [4]			
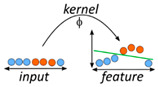	A boundary is determined that separates the classes. For nonlinear separation, the data is augmented by additional dimensions using a kernel function (Φ), often the Radial Basis Function (RBF).	Features/sample: High Implementation: Easy	Overfitting risk: High Interpretation: low
ANN—Artificial Neural Network [5]			
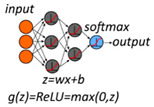	The features are transformed by hidden nodes with a linear equation ‘z’ and a nonlinear function ‘g.’ Several layers may follow, each with nodes containing transformations by functions ‘z’ and ‘g.’ The output is generated by a ‘softmax’ function.	Features/sample: Medium	Overfitting risk: High Interpretation: Medium Implementation: Complex
GA—Genetic Algorithm [6]			
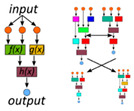	Solution space is searched by operations similar to natural genetic processes to identify suitable solutions. Fitness function is defined to find the fittest solutions. The fittest solutions are subject to cross-over and mutations to evolve towards the best solution.	Interpretation: High	Overfitting risk: High Features/sample: Medium Implementation: Complex

**Table 2:** Available spectral database for metabolite annotation *.

Database	Description	URL
HMDB [29]	114,193 metabolite entries including both polar and non-polar metabolites	https://hmdb.ca
LMSD [30]	43,665 lipid structures with MS/MS spectra	www.lipidmaps.org/data/structure
METLIN [31]	961,829 molecules (lipids, steroids, plant and bacteria metabolites, small peptides, carbohydrates, exogenous drugs/metabolites, central carbon metabolites and toxicants). Over 14,000 metabolites have been individually analyzed and another 200,000 have in silico MS/MS data	http://metlin.scripps.edu
isoMETLIN [32]	All computed isotopologues derived from METLIN based on *m/z* values and specified isotopes of interest (^13^C or ^15^N)	http://isometlin.scripps.edu
NIST [33,34]	Reference mass spectra for GC/MS, LC–MS/MS, NMR and gas-phase retention indices for GC	https://chemdata.nist.gov
MassBank [35]	Shared public repository of mass spectral data with 41,092 spectra	https://massbank.eu/MassBank
MoNA	200,000+ mass spectral records from experimental, in silico libraries and user contributions	https://mona.fiehnlab.ucdavis.edu
mzCloud	More than 6 million multi-stage MS^n^ spectra for more than 17,670 compounds	https://www.mzcloud.org
PRIME [36,37]	Standard spectrum of standard compounds generated by GC/MS, LC–MS, CE/MS and NMR	http://prime.psc.riken.jp/
*Golm metabolome* [38]	2019 metabolites with GC-MS spectra and retention time indices	http://gmd.mpimp-golm.mpg.de
*GNPS* [39]	Community database for natural products	https://gnps.ucsd.edu
*ReSpect* [40]	Over 9000 MS/MS spectrum of phytochemicals	http://spectra.psc.riken.jp

* Adapted and updated from An PNT et al. [41].

**Table 3:** ML tools for data processing since 2019.

Step	ML Tool	Example	Ref.
Peak picking/integration	SVM	WIPP software: optimization of peak detection, instrument and sample specific	[57]
	ANN	Peak quality selection for downstream analysis	[58]
	CNN	Trace: two-dimensional peak picking over retention time and m/z	[59]
	CNN	peakonly software: peak picking and integration	[60]
	CNN	Peak classification for subsequent PARAFAC analysis	[43]
	CNN	DeepSWATH software: correlation between parent metabolites and fragment ions in MS/MS spectra	[61]
	CNN	Representational learning from different tissues, organisms, ionization, instruments for improved classification on small datasets	[62]
	CNN	‘DeepSpectra’: targeted metabolomics on environmental samples, raw spectra analysis	[49]
	CNN	Compound recognition in complex tandem MS data tested with several ML tools	[63]
Retention time prediction	ANN	Metlin-integrated prediction of metabolite retention time extrapolation to different chromatographic methods	[48]
	Ensemble	Performance test of multiple ML algorithms for retention time prediction based on physical properties, ANN and SVM perform well, ensemble training is optimal	[47]
Metabolite annotation	SVM	Input–output kernel regression (IOKR) to predict fingerprint vectors from m/z spectra, mapping molecular structures to spectra	[64]
	SVM	CSI:Fingerprint:Structure mapping	[52]
	Text mining	MS2LDA software: detection of peak co-occurrence	[56]
	Text mining	MESSAR software: automated substructure recommendation for co-occurring peaks	[55]
	ANN	NEIMS software: ‘neural electron-ionization MS’ spectrum prediction	[54]
	ANN	DeepMASS software: substructure detection by comparing unknown spectra to known spectra	[65]
	CNN	DeepEI software: fingerprint prediction from MS spectrum	[54]
Normalization	RF	SERRF software: Systematic error removal based on quality control pool samples	[66]
	RF	pseudoQC software: simulated quality control sample generation, preferably with RF	[67]
	SVM	Improvement of statistical analysis by SVM normalization	[68]
Imputation	RF	Best overall performance of RF for unknown missing value type	[69]
	Bayesian Model	BayesMetab: classification of missing value type, Markov chain Monte Carlo approach with data augmentation	[70]

**Table 4:** Data to knowledge procedures with ML support published from 2019. In some cases, different datasets (DS) are used for samples. Spec-Type—spectrometry type; Ens.—ensemble ML approach; Tar.—targeted; SCMS—single-cell MS; Bench. data—benchmark datasets; Sim.—simulated.

Biological Insight	Optimal ML	Other Models	Samples	Dimension Reduction	Spec-Type	Comment	Ref.
Class + biomarker	SVM	LDA, QDA	4 DS: 30, 280, 240, 183	PCA	IR	Effect of variance and covariance on classification of infrared spectra.	[79]
	SVM	RF, PLS-DA	80	RFE	LC–MS	Serum identification of lipids, glycans, fatty acids.	[80]
	RF	N.A.	<100	N.A.	SCMS	Single-cell MS on drug response, pathway inference.	[81]
	RF	SVM, ANN, CNN	703	LASSO	LC–MS	Serum metabolomics related to chronic kidney disease.	[82]
	RF	N.A.	3 DS: 39, 160, 79	Peak-binning	GCMS	Chromatogram peak ranking for sample discrimination.	[83]
	RF	N.A.	217	Human selection	LC–MS	Metabolite selection based on expert classification with tinderest Shiny-App.	[84]
	ANN	PLS-DA, RF SVM	10 DS: 968, 253, 668, 59, 184, 97, 80, 100, 121, 83	N.A.	Bench. data	Thorough comparison of ML approaches on different published targeted MS datasets.	[78]
	GA	RF	60	N.A.	LC–MS	Wine origin classification.	[85]
	Ens.	RF, SVM	111	Correlation, information filter	N.A.	Use of symbolic methods, analysis of spectrogram.	[86]
Regression	Ens.	RF, ANN	2 DS: 36, 60	N.A.	Assay	Optimization of gene expression for metabolite overproduction.	[87]
Pathway inference	RF	Bayes	500	N.A.	Sim.	Metabolite correlation network on simulated data.	[88]
	RF	PLS, Bayes	339	Information filter	GCMS	Mapping of metabolic correlation networks to metabolic pathways.	[89]
	Bayes	N.A.	2 DS: 8711, 384	N.A.	Sim.	‘PUMA’: Probabilistic modeling for Untargeted Metabolomics Analysis. Simulation of pathway activity, metabolite association, and spectra.	[90]
Multi-omics integration	ANN	SVM	2 DS: 600, >10,000	Encoder-decoder	LC–MS/MS	Multi-omics projection to 20–70 latent variables. Classification of latent variables.	[91]
	ANN	N.A.	2 DS: 191 in: 1692 out, 51 in: 143 out	Encoder-decoder	LC–MS	Correlation of gut bacteria level to metabolite level, unsupervised clustering of latent variables.	[92]
	Text Mining	N.A.	4 DS: 138 in: 462 out, 466 in: 85 out, 902 in: >10k out, 562 in: > 10k out	N.A.	Bench. data	‘mmvec’: microbial sequence to metabolite occurrence mapping with as little as 166 microbes mapped to 85 metabolites	[93]
	Bayes	N.A.	25	N.A	Sim.	Estimation of metabolic kinetics based on multi-omics data for lysine synthesis.	[94]
	Bayes	N.A.	22	N.A.		Estimation of metabolic kinetics based on multi-omics data	[95]

**Table 5:** Dimensionality reduction strategies. FS—feature selection; FE—feature extraction.

Type	Method	Description	Advantages	Disadvantages
Unsupervised method				
FE	Principal Component Analysis (PCA)	Unsupervised method to transform data into axes that explain maximum variability. Returns orthogonal features.	Prior Information: None	Interpretation: Low
FE	Kernel PCA (k-PCA)	Transforms the data into a lower dimension that is linearly separable.	Correlation type: Nonlinear data	Interpretation: Low
FE	Encoder–Decoder	ANN-based, the encoder maps input to lower-dimensional latent variables. The decoder uses latent variables to generate output.	Correlation type: Nonlinear data Prior Information: None	Correlation type: Fails on independent data
Regularization				
FS	LASSO or L1	Supervised method to select sparse features. Regularization parameter (L1 penalty) can be used for regression and classification problems. The coefficients (*w*) of the features (*m*) are directly multiplied with the regularization parameter (*λ*). L1: *λ*$\overset{m}{\underset{k=0}{\sum }}w_{k}$	Interpretation: High	Correlation type: Linear data Note: Minimum selection of features equal to sample size
FS	Ridge or L2	Supervised method to penalize (L2 penalty) large individual weights. The coefficients (*w*) of the features (*m*) are squared and multiplied with the regularization parameter (*λ*). L2: *λ*$\overset{m}{\underset{k=0}{\sum }}w_{k}^{2}$	Note: Avoids overfitting	Note: Features are not removed, weights indicate feature importance
FS	Elastic Net	Regularization method to retain advantages of both L1 and L2 penalty. EN: *λ*_1_$\;\overset{m}{\underset{k=0}{\sum }}w_{k}+$ *λ*_2_ $\overset{m}{\underset{k=0}{\sum }}w_{k}^{2}$	Note: Removes features without overfitting	Correlation type: Linear data
Discriminant Analysis				
FE	Linear Discriminant Analysis (LDA)	Supervised method to transform data into axes, which maximizes class separation. Assumes that data is normal with common class covariance.	Prior information: Class labels	Correlation type: Linear data Interpretation: Low
	Quadratic Discriminant Analysis (QDA)	Supervised classification similar to LDA. Assumes that data is normal but allows for differing class covariance.	Correlation type: Squared nonlinear data	Not useful for dimensionality reduction
Sequential Feature Selection				
FS	Recursive Feature Elimination/Sequential Backward Selection	At each step, the feature with minimal contribution to the model is dropped until required number of features remain.	Interpretation: High	Note: Optimum not guaranteed

**Table 6:** Cross-consistency matrix with categorical research topics of multi-omics integration. M—metabolomics; T—transcriptomics; P—proteomics; F—fluxomics.

		Data					Integration Method			Dimensionality Reduction				Model Organisms			
		MT	MP	MTP	MPF	MTPF	Concatenation	Post-Analysis Integration	Ensemble	PCA	Regularization	LDA	SFE	*Escherichia coli*	*Danio rerio*	*Saccharomyces cerevisiae*	Mammalian
Model	Partial least squares	[131]	[109]	[132]			[131,133]				[132]				[133]	[132]	[131]
	Random forest	[120]	[119]	[132]	[122]		[119]	[118,120]			[132]		[118]			[132]	[118,119,120]
	SVM	[120,121]		[132]			[121]	[120]			[132]		[121]			[132]	[120,121]
	Artificial neural network			[132]		[134]			[134]		[132,134]			[134]		[132]	
	Genetic algorithms		[109]											[109]			
	Bayesian models				[94,95,122]				[95]					[95]		[94,122]	
Data	MT						[121,131,133]	[120]				[120]	[121]		[131]		[120,121]
	MP						[119]	[135]		[135]				[109,135]			[119]
	MTP										[132]					[132]	[136]
	MPF								[122]							[94,122]	
	MTPF							[137]	[134]	[137]	[134]			[134]			
Integration Method	Concatenation												[121]		[131]		[119,121]
	Post-analysis integration									[135,137]		[120]	[118]	[135]			[118,120]
	Ensemble										[134]			[134]			
Dimensionality reduction	PCA													[135]		[137]	
	Regularization													[134]		[132]	
	LDA																[120]
	SFE																[118,121]

## Abbreviations and Terms

Activation Function	The function that defines whether a neuron in a neural network is active.
Bayesian model	Bayes theorem is used with prior probabilities of past events for prediction.
CNN	Convolutional neural networks are a special form of artificial neural networks, strong when feature geometry is important as in images or spectral data.
Cross validation	Data is divided into folds, where every fold is used as a test set and average metrics across the folds are used to evaluate model statistics.
Feature	Observed variable used as input to the model for prediction.
Hyperparameter	Also known as metaparameters and used for tuning of the model training.
Latent variables	Features derived by mathematical transformation of features.
Overfitting	The model performs well on the training data but poorly on unknown data. Overfitting increases with variables and nonlinearity of the statistical model. Cross validation identifies overfitting.
